# Dose escalation of radiation therapy with or without induction chemotherapy for unresectable locally advanced pancreatic cancer

**DOI:** 10.1186/s13014-018-1158-z

**Published:** 2018-11-06

**Authors:** Sung Jun Ma, Kavitha M. Prezzano, Gregory M. Hermann, Anurag K. Singh

**Affiliations:** Department of Radiation Medicine, Roswell Park Comprehensive Cancer Center, Elm and Carlton Streets, Buffalo, NY 14263 USA

**Keywords:** Induction chemotherapy, Locally advanced pancreatic cancer, Dose escalation, Conventionally fractionated

## Abstract

**Background:**

Dose escalation of conventionally fractionated radiation therapy (CFRT) above 45–54 Gy has an unclear survival benefit. Prior National Cancer Database (NCDB) analyses have shown improved overall survival with induction chemotherapy (iC) prior to concurrent chemoradiation (CRT) in locally advanced pancreatic cancer. Our study compared dose-escalated CFRT with and without iC.

**Methods:**

The NCDB was queried for primary stage III, cT4 N0–1 M0 LAPC treated with CRT with or without iC (2004–2015). CFRT was stratified by < 55 Gy and ≥ 55 Gy. Cohort iC + CRT and CRT included those with and without iC, respectively. The primary endpoint was overall survival (OS). Kaplan-Meier analysis, Cox proportional hazards method, and propensity score matching were used.

**Results:**

Among 2029 patients, cohort iC + CRT had 738 patients (*n* = 601 for 45–55 Gy and *n* = 137 for ≥55 Gy) and cohort CRT had 1291 patients (*n* = 1066 for 45–55 Gy and *n* = 225 for ≥55 Gy). Median follow-up was 24.3 months and 24.6 months for cohorts iC + CRT and CRT, respectively. Dose escalation showed improved survival in the multivariable analysis in cohort iC + CRT (HR 0.77, *p* = 0.013) but not in cohort CRT (HR 0.91, *p* = 0.19). Using 2:1 propensity score matching, a total of 387 patients for cohort iC + CRT and 549 patients for cohort CRT were matched. After matching, dose escalation remained significant for improved overall survival in cohort iC + CRT (median OS 16.2 vs 15.2 months; 2-yr OS 33.4% vs 25.4%; *p* = 0.022) but not in cohort CRT (median OS 11.8 vs 10.6 months; 2-yr OS 13.3% vs 10.1%; *p* = 0.16).

**Conclusions:**

Patients with locally advanced pancreatic cancer who undergo iC have improved survival with radiation dose escalation above 55 Gy. For patients without iC, there is no clear association between radiation dose escalation and survival.

## Background

Pancreatic adenocarcinoma is the fourth leading cause of cancer death in the United States with a dismal 5 year survival of 8% [1]. Surgical resection remains the only potential curative approach in the treatment of this disease, though only 20% of patients are initially able to undergo resection [2]. For patients with unresectable pancreatic cancer, induction chemotherapy (iC), concurrent chemoradiation (CRT) and radiation therapy (RT) alone are among the combination of regimens that have been used as various treatment options.

The role of RT in the setting of locally advanced pancreatic cancer is controversial since responses are limited and the predominant cause of death in these patients is distant metastatic disease. More recent evidence from postmortem studies has shown that up to 30% of deaths from pancreatic cancer are due to locally advancing disease, pointing to the importance of local control in preventing tumor progression and potentially improving overall survival [3].

Radiation dose escalation has been posited as a potential strategy to improve outcomes in this challenging patient population, though results have been mixed. A phase II study of RT alone found that dose escalation to 70–72 Gy was feasible [4]. A National Cancer Database (NCDB) review of patients who received definitive CRT for unresectable pancreatic cancer did not find an overall survival (OS) benefit with the use of RT doses exceeding 45 Gy [5]. Institutional reports have found that dose escalation may provide a significant survival benefit when used in conjunction with iC [6–8].

This study used the NCDB to identify patients with non-metastatic, unresected pancreatic cancer in order to evaluate the role of dose-escalated CRT. We further sought to compare the outcomes of dose escalation in those patients who received iC prior to CRT versus those who underwent definitive CRT alone.

## Methods

### Patient population

The NCDB registry was used to identify pancreatic adenocarcinoma cases diagnosed between 2004 and 2015 (the most recent dataset available at the time of this study). The NCDB is a national cancer database capturing approximately 70% of cancer incidences in the United States and obtains data from over 1500 hospitals [9]. It is a de-identified dataset and this study was exempt from institutional review board review.

**Fig. 1:**
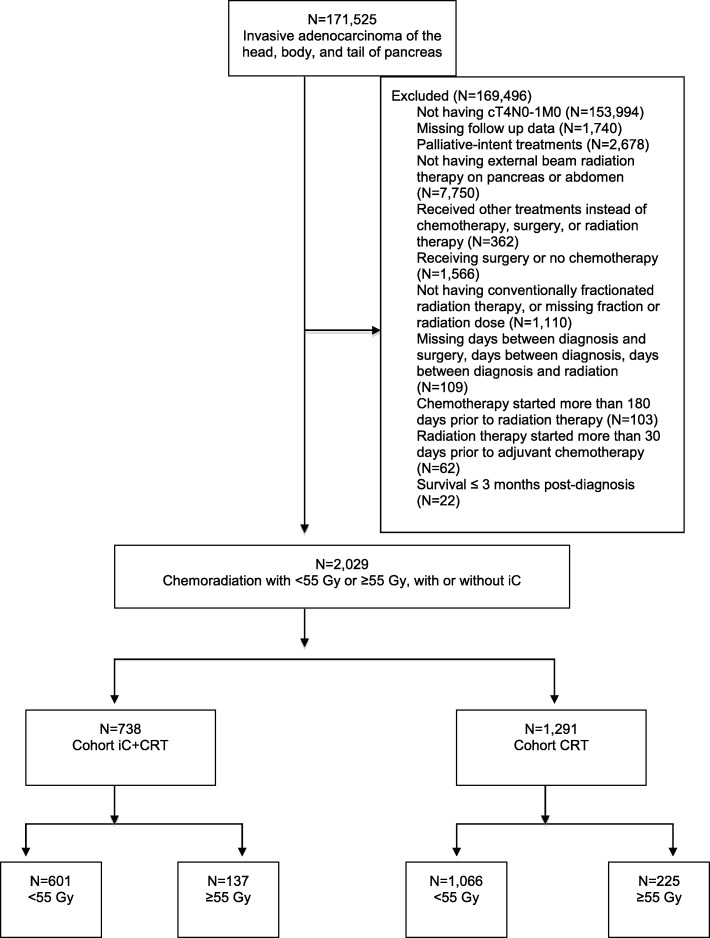
CONSORT diagram for patient selection criteria. iC: induction chemotherapy; CRT: chemoradiation

Our patient selection flow diagram is shown in Fig. 1. Our initial query identified patients with unresected stage III, clinical T4 N0–1 M0 pancreatic adenocarcinoma who had been treated with curative-intent CRT with or without iC. American Joint Committee on Cancer (AJCC) 6th and 7th editions were used to determine stage III disease in 2004–2015.

To address variability in dose fractionation among hospitals for LAPC, conventionally fractionated radiation therapy (CFRT) was categorized as 1.8–2.5 Gy/fractions up to a total of 45–70 Gy [6]. The radiation dose was stratified by < 55 Gy and ≥ 55 Gy based on National Comprehensive Cancer Network (NCCN) guidelines suggesting 45–54 Gy as the standard dose of radiation therapy [10]. Chemotherapy or radiation therapy delivered within 30 days of each other was considered as CRT. Chemotherapy administered within 31–180 days prior to the radiation therapy was considered as iC followed by CRT. Patients who received chemotherapy more than 180 days prior to radiation therapy were excluded from our analysis. Other exclusion criteria were: having undergone surgery, incomplete follow-up, incomplete radiation dose or fractionation, missing data on the number of days between diagnosis and treatments, and palliative-intent treatments. Those who survived less than 3 months after their diagnosis were excluded in order to omit patients who expired prior to completing their treatment course.

Baseline characteristics for analysis included: treatment facility type, age, gender, race, insurance status, household income, residential setting, Charlson-Deyo Score (CDS), year of diagnosis, the location of primary tumor within the pancreas, tumor size, clinical N stage, single- vs multi-agent chemotherapy (defined by the NCDB as the first course of therapy), radiation dose, and radiation fractionation. Cohorts iC + CRT and CRT were constructed to include patients with and without iC, respectively. Age and tumor size were stratified by ≥65 years or < 65 years and < 3.8 cm or ≥ 3.8 cm based on their median values. The household income level of residential area was based on the 2012 American Community Survey data adjusted for inflation (the most recent data at the time of this study), and it was stratified by above or below the median value of $48,000.

Pertinent prognostic factors such as a patient’s performance status, or the type and duration of chemotherapy received are unavailable in the NCDB. Other important outcomes such as toxicity, local and distant recurrences are also unavailable in the NCDB. CA 19–9 factor and tumor grade were missing in 1090 (53.7%) and 1574 (77.6%) patients, respectively. Radiation techniques data for intensity-modulated radiation therapy (IMRT) or conformal 3-D therapy were only available in 457 (61.9%) patients for cohort iC + CRT and 570 (44.2%) patients for cohort CRT. The primary outcome was overall survival (OS), which was characterized as time between the diagnosis and the last follow-up or death.

### Statistical analysis

Kaplan-Meier and log-rank tests were used to evaluate OS. Categorical and continuous variables between the < 55 Gy and ≥ 55 Gy groups were compared using Fisher’s exact and Mann-Whitney U tests, respectively. Logistic regression univariable (UVA) and multivariable analyses (MVA) were performed to identify potential predictors for the dose escalation and were reported as odds ratio (OR). Cox proportional hazard UVA and MVA were performed to identify potential predictors for the OS and were reported as hazards ratio (HR). All statistically significant factors from UVA were used to construct the MVA model, which was finalized using a backward stepwise elimination. Potential treatment interactions with other variables were evaluated using Cox MVA by adding interaction terms. To minimize selection bias, propensity score matching was performed. Matching was based on baseline characteristics, including facility type, age, CDS, year of diagnosis, tumor size, clinical N stage, and single- vs multi-agent chemotherapy. Additional variables were included if they were statistically significant in Cox MVA for OS. All matching was performed in a 2:1 ratio without any replacement, using nearest neighbor method with a caliper distance of 0.2 of the standard deviation of the logit of the propensity score [11]. MatchIt package (version 3.0.1) was used for matching. After matching, matched-sample Cox UVA was used to assess the effect of dose escalation on OS. R software (version 3.4.3, R Foundation for Statistical Computing, Vienna, Austria) was used to perform all aforementioned analyses. All *p* values were two-sided, and the p values less than 0.05 were deemed statistically significant.

## Results

### Cohort iC + CRT

A total of 2029 patients with unresected clinical stage III T4 N0–1 M0 pancreatic adenocarcinoma treated with concurrent chemoradiation were examined. Of those, 738 patients received iC and were included in cohort iC + CRT. Radiation dose of < 55 Gy was delivered to 601 patients, while that of ≥55 Gy was given to 137 patients. The majority of patients in this cohort had clinical T4N0M0 adenocarcinoma of the pancreatic head (Table 1). The patient group treated with ≥55 Gy was more likely to have female patients and fewer patients treated in 2012–2015. Other variables were well balanced.

**Table 1:** Baseline characteristics, before matching

	Cohort iC + CRT					Cohort CRT				
	45–55 Gy		55+ Gy			45–55 Gy		55+ Gy		
	N	%	N	%	P	N	%	N	%	P
Facility					0.39					0.0031
Nonacademic	335	56	71	52		672	63	163	72	
Academic	259	43	65	47		390	37	58	26	
NA	7	1	1	1		4	0	4	2	
Age					1					0.0081
< 65	303	50	69	50		474	44	122	54	
≥ 65	298	50	68	50		592	56	103	46	
NA	0	0	0	0		0	0	0	0	
Gender					0.047					1
Female	297	49	81	59		514	48	109	48	
Male	304	51	56	41		552	52	116	52	
NA	0	0	0	0		0	0	0	0	
Race					0.88					0.18
White	493	82	115	84		869	82	190	84	
Black	81	13	17	12		168	16	27	12	
Other	19	3	3	2		20	2	7	3	
NA	8	1	2	1		9	1	1	0	
Insurance					0.31					0.095
None	12	2	2	1		28	3	7	3	
Nonprivate	320	53	63	46		633	59	117	52	
Private	265	44	70	51		391	37	99	44	
NA	4	1	2	1		14	1	2	1	
Income					1					0.45
Above median	389	65	90	66		546	51	118	52	
Below median	207	34	47	34		503	47	97	43	
NA	5	1	0	0		17	2	10	4	
Residential setting					0.33					0.077
Metro	488	81	114	83		807	76	151	67	
Urban	77	13	16	12		189	18	53	24	
Rural	10	2	5	4		34	3	7	3	
NA	26	4	2	1		36	3	14	6	
Charlson-Deyo Score					1					0.25
0–1	575	96	131	96		1006	94	217	96	
≥ 2	26	4	6	4		60	6	8	4	
NA	0	0	0	0		0	0	0	0	
Year of diagnosis					0.016					< 0.001
2004–2007	52	9	7	5		317	30	92	41	
2008–2011	245	41	74	54		529	50	105	47	
2012–2015	304	51	56	41		220	21	28	12	
NA	0	0	0	0		0	0	0	0	
Primary tumor site					0.068					0.014
Head	411	68	94	69		764	72	140	62	
Body	171	28	43	31		271	25	74	33	
Tail	19	3	0	0		31	3	11	5	
NA	0	0	0	0		0	0	0	0	
Tumor size (cm)					0.70					0.018
< 3.8	265	44	65	47		486	46	82	36	
≥ 3.8	283	47	64	47		448	42	111	49	
NA	53	9	8	6		132	12	32	14	
Clinical N stage					0.44					0.70
0	367	61	89	65		692	65	143	64	
1	234	39	48	35		374	35	82	36	
NA	0	0	0	0		0	0	0	0	
Chemotherapy					0.20					0.74
Single agent	166	28	30	22		789	74	164	73	
Multi agent	435	72	107	78		277	26	61	27	
NA	0	0	0	0		0	0	0	0	
Total radiation dose (Gy)					< 0.001					< 0.001
Median	50.4		59.4			50.4		59.4		
IQR	50.4–52.5		56.0–59.4			50.4–52.0		57.5–60.0		
Fraction					< 0.001					< 0.001
Median	28		30			28		32		
IQR	27–28		28–33			26–28		30–33		

*iC* induction chemotherapy, *CRT* chemoradiation, *IQR* interquartile range, *NA* not available

On logistic regression UVA, male patients were less likely to receive ≥55 Gy (OR 0.68, *p* = 0.041). No other variables were statistically significant for the receipt of dose escalation.

On Cox MVA (Table 2), having treatments at academic facilities (HR 0.79, *p* = 0.0045), diagnosis between the years 2012–2015 (HR 0.71, *p* = 0.021), the use of multi-agent chemotherapy (HR 0.77, *p* = 0.0041), and dose escalation ≥55 Gy (HR 0.77, *p* = 0.013) were associated with improved survival. After Cox MVA, there was no treatment interaction with age ≥ 65 vs < 65 (*p* = 0.54), CDS ≥2 vs 0–1 (*p* = 0.90), year of diagnosis (2008–2011, *p* = 0.93, 2012–2015, *p* = 0.89), tumor size ≥3.8 cm vs < 3.8 cm (*p* = 0.37), or pancreatic tumor site (body and tail, *p* = 0.18). The ideal period of induction chemotherapy was identified using a restricted cubic spline method, which demonstrated HR 1.0 crossing at approximately 91 days. Therefore, two patient population groups were constructed (*n* = 360 for < 90 days, *n* = 378 for ≥90 days). Longer duration of induction chemotherapy remained statistically significant for improved overall survival in multivariable analysis (HR 0.83, *p* = 0.025).

**Table 2:** Cox UVA and MVA for cohort iC + CRT

Variable	Cox UVA			Cox MVA		
	HR	95% CI	P	HR	95% CI	P
Facility						
Nonacademic	1	Ref		1	Ref	
Academic	0.76	0.65–0.89	< 0.001	0.79	0.67–0.93	0.0045
Age						
< 65	1	Ref				
≥ 65	1.04	0.89–1.21	0.64			
Gender						
Female	1	Ref		1	Ref	
Male	1.19	1.02–1.39	0.031	1.09	0.93–1.29	0.29
Race						
White	1	Ref				
Black	0.86	0.68–1.10	0.23			
Other	0.86	0.54–1.36	0.52			
Insurance						
None	1	Ref				
Nonprivate	1.07	0.57–2.02	0.82			
Private	1.07	0.57–2.01	0.83			
Income						
Above median	1	Ref				
Below median	1.05	0.90–1.24	0.52			
Residential setting						
Metro	1	Ref				
Urban	1.00	0.79–1.26	0.98			
Rural	1.35	0.80–2.25	0.26			
Charlson-Deyo Score						
0–1	1	Ref				
≥ 2	1.08	0.75–1.57	0.67			
Year of diagnosis						
2004–2007	1	Ref		1	Ref	
2008–2011	0.95	0.71–1.28	0.75			
2012–2015	0.67	0.50–0.89	0.0068	0.71	0.53–0.95	0.021
Primary tumor site						
Head	1	Ref				
Body	0.87	0.73–1.04	0.12			
Tail	1.25	0.78–2.01	0.35			
Tumor size (cm)						
< 3.8	1	Ref		1	Ref	
≥ 3.8	1.20	1.02–1.41	0.027	1.16	0.98–1.37	0.082
Clinical N stage						
0	1	Ref				
1	1.15	0.98–1.34	0.091			
Chemotherapy						
Single agent	1	Ref		1	Ref	
Multi agent	0.69	0.58–0.82	< 0.001	0.77	0.64–0.92	0.0041
Dose escalation						
< 55 Gy	1	Ref		1	Ref	
≥ 55 Gy	0.80	0.65–0.97	0.027	0.77	0.63–0.95	0.013

*UVA* univariate analysis, *MVA* multivariable analysis, *HR* hazard ratio, *CI* confidence interval, *Ref* reference

The overall median follow-up in cohort iC + CRT was 24.3 months (interquartile range [IQR] 16.2–38.0). The < 55 Gy group had a median follow-up of 22.8 months (IQR 15.5–33.6) and the ≥55 Gy group had that of 29.6 months (IQR 22.0–54.2). The median OS was 15.9 months (IQR 11.1–22.4) for the < 55 Gy group and 16.4 months (IQR 12.0–26.5) for the ≥55 Gy group (log-rank *p* = 0.026). OS at 2 years was 25.1% for the < 55 Gy group and 35.2% for the ≥55 Gy group.

**Fig. 2:**
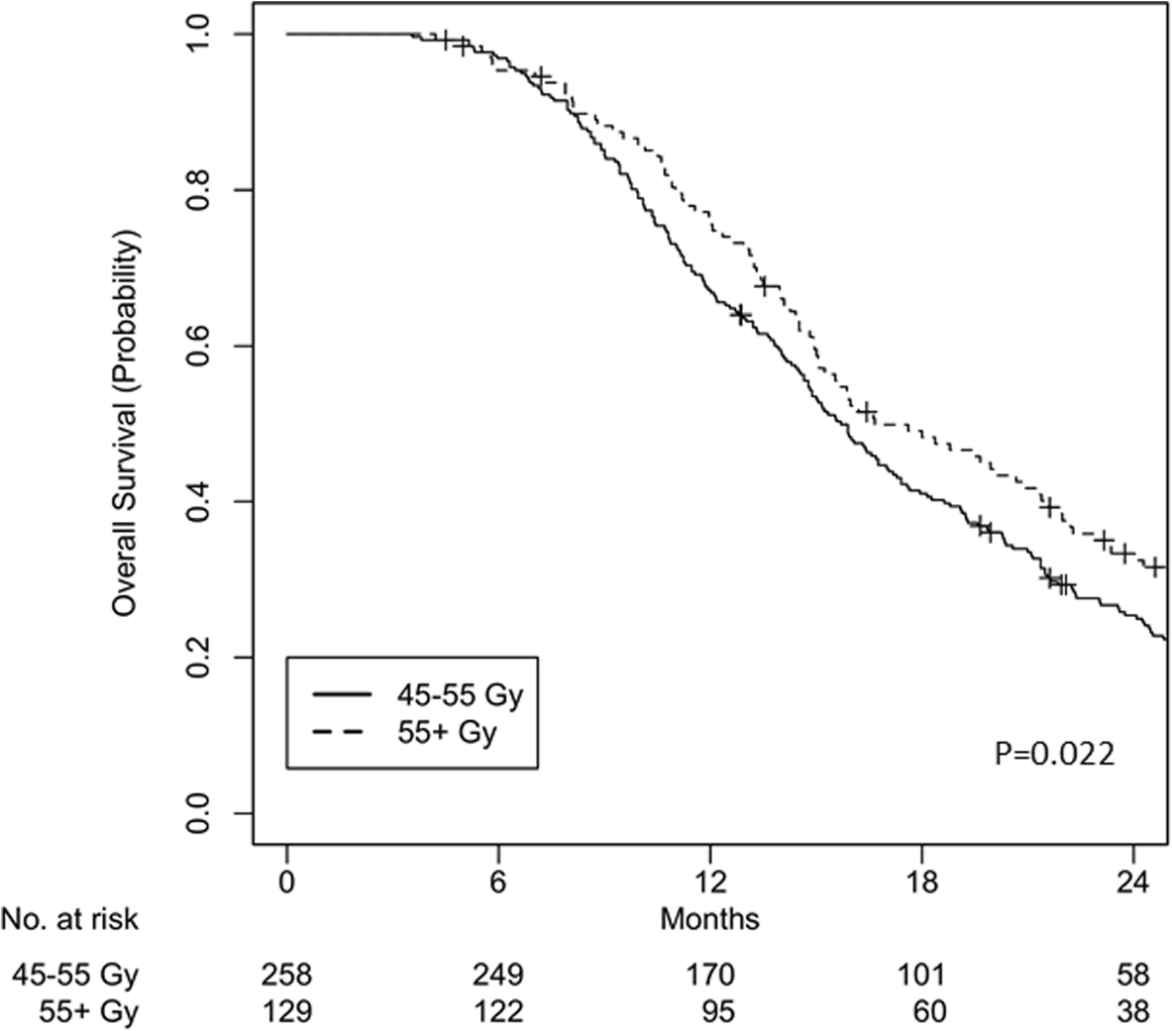
Overall survival for cohort iC + CRT, after matching

A total of 387 patients were matched, with 258 patients in the < 55 Gy group and 129 patients in the ≥55 Gy group. All variables were well balanced between these groups (Table 3). The overall median follow-up for the matched patients was 24.8 months (IQR 16.5–42.7). The median OS was 15.2 months (IQR 10.4–22.3) for the < 55 Gy group and 16.2 months (IQR 12.0–25.3) for the ≥55 Gy group (log-rank *p* = 0.022). OS at 2 years was 25.4% for the < 55 Gy group and 33.4% for the ≥55 Gy group (Fig. 2).

**Table 3:** Baseline characteristics, after matching

	Cohort iC + CRT					Cohort CRT				
	45–55 Gy		55+ Gy			45–55 Gy		55+ Gy		
	N	%	N	%	P	N	%	N	%	P
Facility1										0.26
Nonacademic	129	50	65	50		273	75	128	70	
Academic	129	50	64	50		93	25	55	30	
Age					0.91					0.59
< 65	128	50	65	50		196	54	93	51	
≥ 65	130	50	64	50		170	46	90	49	
Charlson-Deyo Score					0.65					0.48
0–1	242	94	123	95		351	96	178	97	
≥ 2	16	6	6	5		15	4	5	3	
Income										0.65
Above median						205	56	107	58	
Below median						161	44	76	42	
Year of diagnosis					0.71					0.85
2004–2007	10	4	5	4		133	36	71	39	
2008–2011	131	51	71	55		177	48	86	47	
2012–2015	117	45	53	41		56	15	26	14	
Tumor size (cm)					0.67					0.78
< 3.8	137	53	65	50		161	44	78	43	
≥ 3.8	121	47	64	50		205	56	105	57	
Clinical N stage					0.58					0.51
0	160	62	84	65		224	61	118	64	
1	98	38	45	35		142	39	65	36	
Chemotherapy					0.59					0.54
Single agent	49	19	28	22		273	75	132	72	
Multi agent	209	81	101	78		93	25	51	28	

*iC* induction chemotherapy, *CRT* chemoradiation

### Cohort CRT

A total of 1291 patients received no iC and were included in cohort CRT. A radiation dose of < 55 Gy was delivered to 1066 patients, while that of ≥55 Gy was given to 225 patients. Similar to the cohort one, the majority of patients had clinical T4N0M0 adenocarcinoma of the pancreatic head (Table 1). The ≥55 Gy group was more likely to have patients treated at nonacademic facilities, age < 65, diagnosis between 2004 and 2007, body of pancreas as a primary site, and larger tumors.

On logistic MVA, patients with treatments at academic facilities (OR 0.65, *p* = 0.015) and a diagnosis between 2012 and 2015 (OR 0.49, *p* = 0.0037) were less likely to receive ≥55 Gy. In contrast, a larger tumor (OR 1.52, *p* = 0.010) was associated with the receipt of ≥55 Gy.

On Cox MVA (Table 4), age ≥ 65 (HR 1.18, *p* = 0.0055) and lower income (HR 1.21, *p* < 0.001) were associated with higher mortality. In contrast, the use of multi-agent chemotherapy was associated with improved survival (HR 0.81, *p* = 0.0016). On Cox UVA, the association between dose escalation and survival was not statistically significant (HR 0.91, *p* = 0.19) and was excluded from the Cox MVA model. After Cox MVA, no treatment interaction was observed with age ≥ 65 vs < 65 (*p* = 0.27), CDS ≥2 vs 0–1 (*p* = 0.93), year of diagnosis (2008–2011, *p* = 0.99; 2012–2015, *p* = 0.73), tumor size ≥3.8 cm vs < 3.8 cm (*p* = 0.75), or pancreatic tumor site (body and tail, *p* = 0.74).

**Table 4:** Cox UVA and MVA for cohort CRT

Variable	Cox UVA			Cox MVA		
	HR	95% CI	P	HR	95% CI	P
Facility						
Nonacademic	1	Ref		1	Ref	
Academic	0.89	0.79–0.997	0.045	0.91	0.81–1.03	0.14
Age						
< 65	1	Ref		1	Ref	
≥ 65	1.18	1.06–1.32	0.0038	1.18	1.05–1.32	0.0055
Gender						
Female	1	Ref				
Male	1.05	0.94–1.17	0.44			
Race						
White	1	Ref				
Black	0.99	0.85–1.16	0.91			
Other	0.99	0.67–1.48	0.98			
Insurance						
None	1	Ref				
Nonprivate	1.33	0.94–1.88	0.11			
Private	1.09	0.77–1.55	0.63			
Income						
Above median	1	Ref		1	Ref	
Below median	1.21	1.08–1.36	< 0.001	1.21	1.08–1.36	< 0.001
Residential setting						
Metro	1	Ref				
Urban	1.02	0.88–1.17	0.83			
Rural	1.14	0.83–1.56	0.43			
Charlson-Deyo Score						
0–1	1	Ref				
≥ 2	1.01	0.78–1.30	0.94			
Year of diagnosis						
2004–2007	1	Ref				
2008–2011	0.92	0.81–1.04	0.19			
2012–2015	0.91	0.77–1.07	0.25			
Primary tumor site						
Head	1	Ref				
Body	0.96	0.85–1.09	0.57			
Tail	1.04	0.76–1.43	0.80			
Tumor size (cm)						
< 3.8	1	Ref				
≥ 3.8	1.08	0.96–1.21	0.22			
Clinical N stage						
0	1	Ref				
1	1.09	0.97–1.22	0.17			
Chemotherapy						
Single agent	1	Ref		1	Ref	
Multi agent	0.79	0.70–0.90	< 0.001	0.81	0.71–0.92	0.0016
Dose escalation						
< 55 Gy	1	Ref				
≥ 55 Gy	0.91	0.78–1.05	0.19			

*UVA* univariate analysis, *MVA* multivariable analysis, *HR* hazard ratio, *CI* confidence interval, *Ref* reference

The overall median follow-up was 24.6 months (IQR 9.8–51.4) in cohort CRT. The median follow-up was 22.5 months (IQR 8.5–47.6) for the < 55 Gy group and 30.8 months (IQR 22.6–68.1) for the ≥55 Gy group. The median OS of the < 55 Gy group was 10.8 months (IQR 7.1–16.9) and that of the ≥55 Gy group was 11.6 months (IQR 7.6–18.1, log-rank *p* = 0.18). OS at 2 years was 11.3% for the < 55 Gy group and 13.7% for the ≥55 Gy group.

**Fig. 3:**
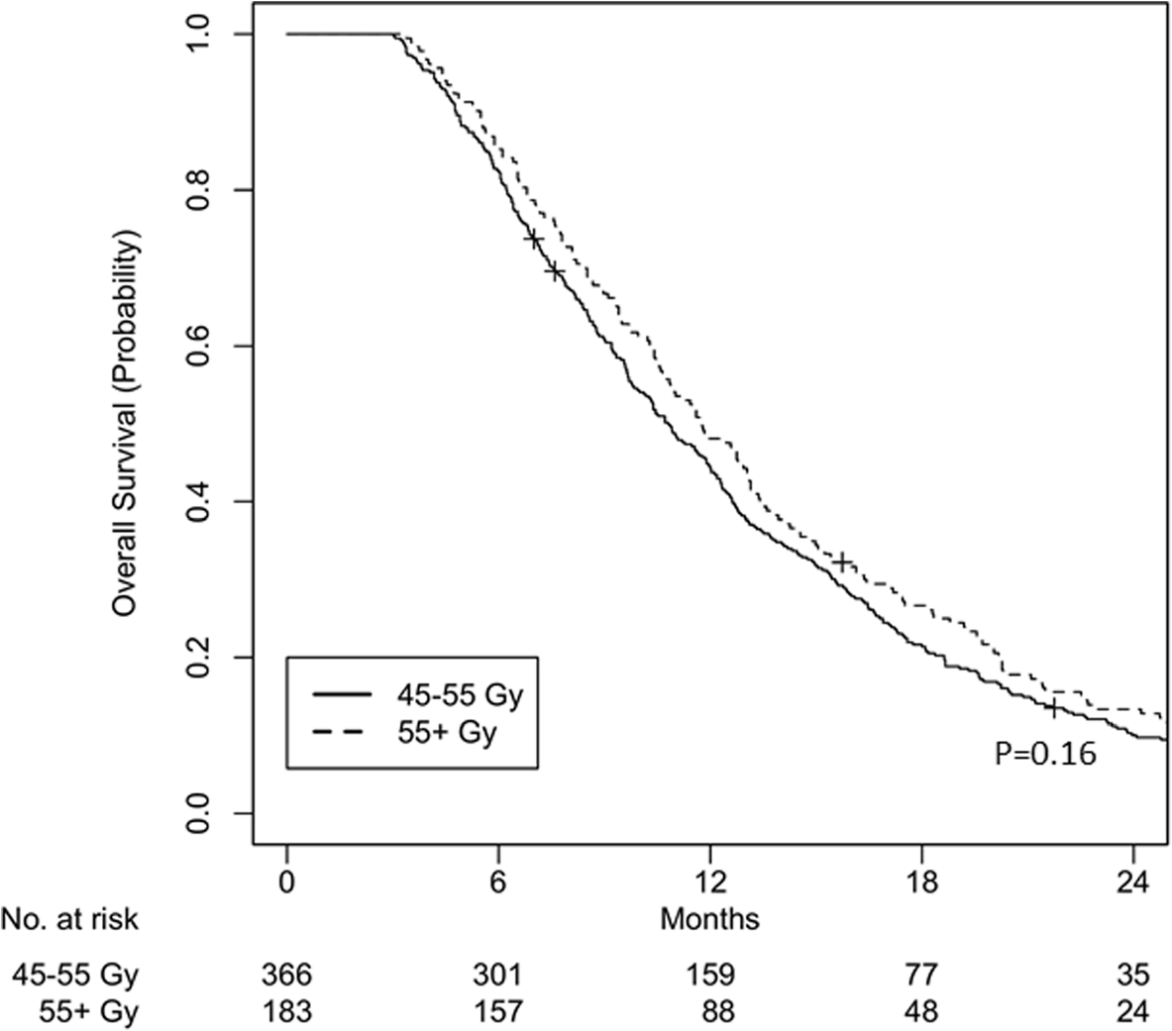
Overall survival for cohort CRT, after matching

A total of 549 patients were matched, with 366 patients in the < 55 Gy group and 183 patients in the ≥55 Gy group. All variables were well balanced (Table 3). The overall follow-up for the matched patients was 27.9 months (IQR 11.2–55.2). The median OS of the < 55 Gy group was 10.6 months (IQR 6.8–16.7) and that of the ≥55 Gy group was 11.8 months (IQR 7.7–18.3, log-rank *p* = 0.16). OS at 2 years was 10.1% for the < 55 Gy group and 13.3% for the ≥55 Gy group (Fig. 3).

### Total cohort

3D conformal radiation therapy (3D-CRT) was delivered to 269 patients (*n* = 192 without induction chemotherapy, *n* = 77 with induction chemotherapy) and intensity-modulated radiation therapy (IMRT) was delivered to 690 patients (*n* = 378 without induction chemotherapy, *n* = 312 with induction chemotherapy). For all patients, 3D-CRT was not associated with worse mortality (HR 1.05, 95% CI 0.91–1.22, *p* = 0.50) compared to IMRT. Table 5 shows COX UVA/MVA for the total cohort and demonstrates iC improves OS (HR 0.73, *p* < 0.001) in the multivariable analysis.

**Table 5:** Cox UVA and MVA for the total cohort

Variable	Cox UVA			Cox MVA		
	HR	95% CI	P	HR	95% CI	P
Facility						
Nonacademic	1	Ref		1	Ref	
Academic	0.80	0.73–0.88	< 0.001	0.90	0.81–0.99	0.036
Age						
< 65	1	Ref		1	Ref	
≥ 65	1.15	1.05–1.26	0.0027	1.18	1.07–1.30	0.0013
Gender						
Female	1	Ref		1	Ref	
Male	1.11	1.01–1.22	0.024	1.08	0.98–1.19	0.13
Race						
White	1	Ref				
Black	0.96	0.85–1.10	0.58			
Other	0.89	0.66–1.20	0.43			
Insurance						
None	1	Ref				
Nonprivate	1.20	0.88–1.62	0.25			
Private	1.02	0.75–1.38	0.91			
Income						
Above median	1	Ref		1	Ref	
Below median	1.22	1.11–1.34	< 0.001	1.20	1.08–1.32	< 0.001
Residential setting						
Metro	1	Ref				
Urban	1.07	0.95–1.21	0.27			
Rural	1.25	0.96–1.64	0.10			
Charlson-Deyo Score						
0–1	1	Ref				
≥ 2	1.06	0.86–1.30	0.61			
Year of diagnosis						
2004–2007	1	Ref		1	Ref	
2008–2011	0.85	0.76–0.95	0.0045	0.92	0.81–1.05	0.20
2012–2015	0.62	0.55–0.71	< 0.001	0.77	0.66–0.89	< 0.001
Primary tumor site						
Head	1	Ref				
Body	0.92	0.83–1.01	0.088			
Tail	1.13	0.87–1.48	0.35			
Tumor size (cm)						
< 3.8	1	Ref		1	Ref	
≥ 3.8	1.11	1.01–1.22	0.035	1.15	1.04–1.27	0.0049
Clinical N stage						
0	1	Ref				
1	1.08	0.99–1.19	0.098			
Chemotherapy						
Single agent	1	Ref		1	Ref	
Multi agent	0.63	0.57–0.69	< 0.001	0.78	0.69–0.87	< 0.001
Induction chemotherapy						
No	1	Ref		1	Ref	
Yes	0.58	0.53–0.64	< 0.001	0.73	0.65–0.83	< 0.001
Dose escalation						
< 55 Gy	1	Ref		1	Ref	
≥ 55 Gy	0.86	0.76–0.97	0.012	0.87	0.76–0.99	0.029

*UVA* univariate analysis, *MVA* multivariable analysis, *HR* hazard ratio, *CI* confidence interval, *Ref* reference

## Discussion

This NCDB analysis sought to examine the effect of radiation dose escalation in patients with locally advanced pancreatic cancer when given concurrently with chemotherapy. To our knowledge, this is the first study that compares the effect of dose escalation in patients who have received iC versus those who received CRT alone. We show that there is a significant OS benefit with the use of dose-escalated RT with concurrent chemotherapy in patients who have first received iC versus those who receive dose-escalated radiation with concurrent chemotherapy alone.

The use of CRT in the setting of unresectable pancreatic cancer is still controversial. The landmark LAP-07 trial, which randomized patients to CRT compared to chemotherapy alone did not demonstrate an improvement in survival, calling into question the utility of RT in locally advanced pancreatic cancer [12]. However, this trial was criticized for its randomization of a heterogeneous cohort of patients, as well as for its use of obsolete chemotherapeutic regimens, and historical radiation techniques. Retrospective studies have reported a survival benefit for patients receiving multi-agent induction chemotherapy followed by CRT [13–15]. A recent NCDB analysis similarly showed that maximal systemic chemotherapy prior to CRT improved survival in patients with unresectable, non-metastatic pancreatic cancer [16]. Interestingly, the Johns Hopkins experience indicated that patients receiving > 2 cycles of iC had decreased progression and trended toward better OS [15]. A recent NCDB analysis similarly showed that maximal systemic chemotherapy prior to CRT improved survival in patients with unresectable, non-metastatic pancreatic cancer, which is consistent with our analysis [16]. Our study adds that in the setting of the maximal systemic chemotherapy prior to CRT, an improved local control with dose-escalated CRT may be translated to an improved OS.

Optimization of the sequencing of chemotherapy and CRT has been explored and is also controversial. A recent investigation by Chung et al. showed a survival advantage in patients receiving a total RT dose ≥61 Gy with concurrent chemotherapy followed by maintenance chemotherapy [7]. However, the FFCD/SFRO (Fédération Francophone de Cancérologie Digestive/Société Francophone de Radiothérapie Oncologique) phase III trial compared induction chemoradiation to chemotherapy alone, followed by maintenance chemotherapy for all patients with locally advanced, unresectable pancreatic cancer [17]. They showed that patients receiving the more intensive induction chemoradiation had worse survival than those receiving chemotherapy alone [17]. This result was corroborated by the Johns Hopkins experience indicating that induction chemotherapy may sensitize tumors to subsequent chemoradiation [15]. It may also be hypothesized from these data that upfront chemotherapy can affect early micrometastatic disease, allowing for increased efficacy of—and improved overall outcomes with—subsequent local therapy.

Based on the FFCD/SFRO study and the Johns Hopkins experience, our finding that patients who underwent iC had a significant survival benefit with dose escalated RT when compared to those who received CRT alone may be explained by the reduction in distant failures with the use of induction chemotherapy [15, 17]. Better local control may then translate into improved OS. Just as in the positive retrospective studies of multi-agent chemotherapy, so too did we find on Cox MVA that multi-agent chemotherapy was associated with improved survival in both cohorts [13, 14, 16]. Since distant failure remains a significant concern in this patient population, any local control benefit that dose escalation may have provided could have been negated in the cohort of patients that did not receive iC. This may explain why dose escalation was ineffective in the absence of iC, but it was effective when used along with iC in prior studies [5, 6]. This hypothesis cannot be validated in our study since the NCDB does not include data on local or distant failure rates, though it is notable that both cohorts included approximately two-thirds of patients with clinical node-negative (N0) disease, suggesting the presence of micro-metastatic disease may be effectively treated by iC. Of course, this does not account for the presumption that patients who received iC were better performers and were able to receive more aggressive therapy.

The SCALOP-2 (Systemic Therapy and Chemoradiation in Advanced Localised Pancreatic Cancer-2) trial is currently enrolling patients with locally advanced, non-metastatic pancreatic cancer on a multi-center, randomized trial evaluating iC followed by standard-dose or dose-escalated radiotherapy with concurrent chemotherapy (ClinicalTrials.gov Number NCT02024009). The results of this study will provide valuable information regarding the benefits and risks associated with intensified local therapies.

Our logistic MVA showed larger tumors were more likely treated with dose-escalated CRT, while treatments in an academic facility were less likely to involve dose escalation. These associations are consistent with a prior NCDB study [5]. Our Cox MVA showed treatments at academic facilities and recently diagnosed patients in 2012–2015 were associated with improved survival, whereas elderly age was associated with worse mortality. These are consistent with previous studies, and treatments at academic facilities with survival benefits may be due to treatment volume [6, 18, 19]. Recent diagnosis with improved survival outcomes in our cohort iC + CRT may be due to the recent adoption of FOLFIRINOX (5-fluorouracil, leucovorin, irinotecan, and oxaliplatin) as iC after the publication of the phase III trial by Conroy et al. in 2011 [18, 20].

A number of limitations, inherent to any retrospective review, are present in this study. Lack of information regarding chemotherapy regimens prevents a more nuanced stratification of patients. Further, data regarding radiation techniques (3D-CRT or IMRT) was limited in many patients. Nonetheless, we included a comparison of 3D-CRT and IMRT, which showed there was no detriment in survival for the delivery of 3D-CRT. This is important, since recent evaluations have shown superior dosimetric constraints and decreased patient morbidity with the use of IMRT, suggesting that there may be an improvement in outcomes with the use of more conformal RT techniques. However our findings are not consistent with a survival advantage, which may be a result of confounding factors within the group of patients whose radiation technique was coded as “unknown” [21–23]. Performance status is not recorded by the NCDB and was therefore unable to be accounted for in this study. Performance status has been shown to be a prognostic factor for overall survival in patients with pancreatic cancer [24], though various studies have also reported that its prognostic significance may be lost in multi-variate analysis [25, 26]. Nevertheless, patients included in this study who were deemed fit enough to tolerate iC were likely to be better performers, and the lack of this information is a confounder on our interpretation. Moreover, the receipt of more aggressive therapy (such as iC and dose escalated RT) would presumably improve outcomes. As part of our methodology, we excluded patients who survived less than 3 months after their diagnosis in order to omit patients who would have been unlikely to complete their course of treatment. Of note, no patients who were excluded for survival less than 3 months, received dose-escalated RT. While this may have unintentionally excluded patients who had increased toxicities from their treatment course, our suspicion that this small number of excluded patients would have suffered acute or fatal toxicities from dose escalation was relatively low, based on several studies [6, 7].

Dose-escalated RT is limited by normal tissue dose constraints, which can result in significant acute and late toxicities. Specifically, tumors in close proximity to stomach and duodenum may limit the amount of RT that could be safely delivered, due to concerns for radiation-induced gastrointestinal toxicities. In this study, no treatment interaction was seen in patients with a tumor size or with tumors located at the body or tail of the pancreas. These variables were also statistically insignificant for survival outcomes after Cox MVA in our study, indicating that they less likely play a major role in the survival outcome of dose escalation seen in both cohorts. Specific toxicities in the two cohorts could not be assessed in this study, since this data is not collected by the NCDB.

The database was also re-examined to evaluate 848 patients who had CA 19–9 factor values, ranging from 0 to > 98. Among 398 patients with no iC, only 47 patients received escalated dose-escalated RT. The sample size of the cohorts with escalated radiation dose was felt to be too small for repeating propensity scored matching. Among 450 patients with induction chemotherapy, only 92 patients received dose-escalated RT. With 1:1 propensity score matching of baseline characteristics including CA 19–9 values, a total of 174 patients were matched. Other ratios such as 2:1 could not be performed since not all patients (55+ Gy) were matched with corresponding, control patients (45–55 Gy). Patients with dose-escalated RT were associated with improved overall survival (log-rank *p* = 0.013).

## Conclusion

This novel NCDB analysis of patients with unresectable, cT4 N0–1 pancreatic cancer shows that dose-escalated RT with concurrent chemotherapy improves survival in patients who have received iC. Use of dose-escalated RT in this setting may be the favored option for definitive treatment of unresectable pancreatic cancer, and further studies may be warranted for this challenging patient population.

## Abbreviations

AJCC: American Joint Committee on Cancer
CDS: Charlson-Deyo Score
CFRT: conventionally fractionated radiation therapy
CRT: concurrent chemoradiation
FFCD: Fédération Francophone de Cancérologie Digestive
FOLFIRINOX: 5-fluorouracil, leucovorin, irinotecan, and oxaliplatin
HR: hazards ratio
iC + CRT: induction chemotherapy followed by concurrent chemoradiation
iC: induction chemotherapy
IMRT: intensity-modulated radiation therapy
MVA: multivariable analyses
NCCN: National Comprehensive Cancer Network
NCDB: National Cancer Database
NCDB: National Cancer Database
OR: odds ratio
OS: overall survival
RT: radiation therapy
SFRO: Société Francophone de Radiothérapie Oncologique
UVA: univariable analysis
